# Cut-off points of the Ishii test to diagnosing severe sarcopenia among multi-ethnic middle-aged to older adults: results from the West China Health and Aging Trend study

**DOI:** 10.3389/fmed.2023.1176128

**Published:** 2023-06-22

**Authors:** Shuyue Luo, Xiaoyan Chen, Lisha Hou, Jirong Yue, Xiaolei Liu, Xin Xia, Li Cao, Birong Dong

**Affiliations:** ^1^National Clinical Research Center for Geriatrics, West China Hospital, Sichuan University, Chengdu, China; ^2^Center of Gerontology and Geriatrics, West China Hospital, Sichuan University, Chengdu, China; ^3^Zigong Psychiatric Research Center, Zigong Affiliated Hospital of Southwest Medical University, Zigong, China

**Keywords:** severe sarcopenia, Western China, Ishii test, AWGS2019, WCHAT study

## Abstract

**Objective:**

This study was designed to establish the cut-off value and diagnostic utility of the Ishii test, which gauges the odds of severe sarcopenia based on the results of an equation based upon age, grip strength, and calf circumference among middle-aged and older adults in Western China.

**Methods:**

This study incorporated adults ≥ 50 years of age from the West China Health and Aging Trend (WCHAT) study. Severe sarcopenia was defined as per the Asian Working Group for Sarcopenia: 2019 Consensus (AWGS2019) recommendations, with the odds of severe sarcopenia being estimated with the Ishii test score chart. The diagnostic utility of the Ishii test in this patient cohort was assessed by analyzing its sensitivity, specificity, positive predictive value (PPV), negative predictive value (NPV), and the area under the ROC curve (AUC).

**Results:**

In total, 4,177 individuals ≥ 50 years of age were included in this study including 2668 females (63.9%) and 1,509 males (36.1%). These included 568 (13.6%) participants affected by severe sarcopenia, of whom 237 were male (15.7%) and 331 were female (12.4%). Optimal Ishii test cut-off values established based on Youden’s index were ≥ 114 for males and ≥ 120 for females when using the AWGS2019 reference standard. The sensitivity/specificity/PPV/NPV of the Ishii test when screening for severe sarcopenia were 89.45%/77.15%/0.42/0.98 in males and 90.03%/77.05%/0.36/0.98 in females. The AUC values for the Ishii test in males and females were 0.899 (95% CI, 0.883–0.916) and 0.905 (95% CI, 0.892–0.917), respectively.

**Conclusion:**

These data indicate that the Ishii test offers value as a candidate diagnostic test that can be used to screen for severe sarcopenia, with recommended diagnostic cut-off values of ≥ 114 for males and ≥ 120 for females.

## Introduction

Severe sarcopenia is a condition associated with a loss of muscle strength and low muscle mass together with altered physical performance as per the Asian Working Group for Sarcopenia: 2019 Consensus (AWGS2019) consensus criteria (1). In one recent meta-analysis of 34 studies, severe sarcopenia prevalence was estimated to range anywhere from 2% to 9% (2). Moreover, severe sarcopenia represents an independent predictor of overall survival, quality of life, and depression. Given its adverse association with multiple different outcomes (3–11), sarcopenia has been a geriatric syndrome of growing focus in recent years. It is thus critical that severe sarcopenia be recognized as quickly as possible in order to guide efforts to mitigate its potentially severe effects.

Several sarcopenia-focused international groups have agreed that a diagnosis of severe sarcopenia necessitates evidence of low muscle strength, low muscle mass, and/or poor physical performance (1, 12–16). Specialized techniques including computed tomography (CT), magnetic resonance imaging (MRI), dual-energy X-ray analysis (DXA), and bio-impedance analysis (BIA) are necessary to definitively diagnose severe sarcopenia. These instruments, however, are costly and require specialized personnel to operate them, restricting their utilization in many medical institutions. Given the lack of access to these diagnostic instruments in some clinical settings, this may lead to the pronounced underdiagnosis of sarcopenia. There is thus a vital need for the design of straightforward, inexpensive, and easy-to-operate tools that can be used to screen for severe sarcopenia in a community setting. Accordingly, the Ishii test was recommended as one such rapid, easy-to-use screening tool by updated version of the European Working Group on Sarcopenia in Older People (EWGSOP2) (17).

The Ishii test was designed by Ishii et al. as a tool that can assess the odds of sarcopenia based on calculations that take patient age, grip strength, and calf circumference into consideration (18). Prior work has demonstrated that Ishii test with the recommended cut-off of 105 for male and 120 for female offers high levels of both sensitivity and specificity when diagnosing sarcopenia among community-dwelling adults and inpatients (18–20). Notably, the Ishii test scores are also significantly related to poorer overall functional status (21), and can predict long-term all-cause mortality among hospitalized older adults (8). A previous study in nursing homes in China showed that the Ishii test be able to differentiate between non-severe and severe sarcopenia with a cut-off of 130 for both male and female (22). No studies to date, however, have examined the cut-off points and accuracy of the Ishii test for male and female separately when used to detect severe sarcopenia in community-dwelling individuals in western China, nor in middle-aged to older adults of various ethnicities in other Asian countries. As such, this study was designed to gauge the cut-off points and accuracy of the Ishii test in both male and female so as to establish whether it can effectively be used to screen for severe sarcopenia among middle-aged and older adults in a community setting so as to provide a foundation for the updating of Asian guidelines as appropriate.

## Materials and methods

### Study design and patient recruitment

All data used herein are from the prospective observational West China Health and Aging Trend (WCHAT) study, which was designed to assess healthy aging among community-dwelling adults in Western China ≥ 50 years of age (23). This study was conducted in accordance with the Declaration of Helsinki, and was reviewed and approved by the Medical Ethics Review Committee of West China Hospital in Sichuan University, Chengdu, Sichuan Province, China (reference: 2017-445). All participants or their appropriate proxy respondents provided written informed consent before study participation. The WCHAT study consisted of three primary components: (1) a questionnaire survey; (2) physical examinations; and (3) laboratory analyses. This study employed a multi-stage random cluster sampling approach covering the Yunnan, Xinjiang, Guizhou, and Sichuan provinces in Western China, and had a 50.2% overall response rate (23). Baseline cross-sectional data were collected from July–December 2018 through questionnaires administered by trained personnel in face-to-face interviews. Anthropometric and BIA measurements were made by qualified personnel. For further details regarding the WCHAT study, see previously published cohort profile information (23). Overall, the WCHAT study incorporated 7,536 individuals who were ≥50 years old in 2018 hailing from 4 provinces and 18 ethnic groups. Participants that did not participate in muscle mass (*n* = 3,036) or for whom data were missing (*n* = 323) were excluded from the present study, with 4,177 participants being retained for analysis.

### Muscle mass measurements

Muscle mass was assessed using a BIA instrument (InBody 770 Body, Seoul, Korea). Both the appendicular skeletal muscle mass (ASM) and the amount of leg and arm muscle were utilized in conjunction with height as a means to determine the ASM index (ASMI; kg/m^2^) (24). Trained personnel completed all measurements within 2 min using the following approach: (1) participants were directed to remove their shoes and to step onto the pedals of the instrument, followed by the input of their personal details; (2) participants remained still in a standing position on the instrument for 5 s such that their weight could be measured; (3) participants gripped the hand electrodes such that four of their fingers were wrapped around the bottom surface of the electrodes while their thumbs were positioned on ovoid electrodes; (4) the participants’ thighs were not allowed to touch, and their heels were aligned with the rear foot electrode; and (5) measurements were initiated when participants gripped the handgrips while maintaining their arms in a straight position opened at a 15° angle. Several precautions were observed when conducting this test, including the following: (1) all testing was performed a minimum of 2 h after eating, and participants were directed to empty their bladder and bowels and to wear light clothing to minimize measurement errors; (2) participants were directed to stand still for 10 min prior to testing to decrease water accumulation in the lower limbs as a result of sudden standing; (3) participants were not permitted to carry any heavy objects or metal items during measurements, including keys, bracelets, watched, and mobile phones; (4) testing was not performed for any individuals with electronic medical devices such as pacemakers; and (5) participants were directed to refrain from talking or moving during the test other than as directed by the personnel administering the testing.

### Muscle strength measurements

A digital grip-strength dynamometer (EH101; Camry, Xiangshan Inc. China) was used to assess participant muscle strength. Subjects were initially questioned regarding which hand was their dominant hand. During testing, they were then directed to grip the dynamometer using their dominant hand while standing upright with their feet shoulder-width apart, allowing their arms to droop naturally. At the start of testing, participants were directed to grip the handle using their full capacity. Participants were not permitted to swing their arms, grip the handle intermittently, squat, or contact the dynamometer using other parts of the body. Grip strength was measured two times, and the maximum value was recorded for downstream analyses.

### Physical performance measurements

A stride meter (TF-NZ1033, Tsinghua Tongfang, China) was used to measure the walking speed of each participant over a 4 m distance to the nearest 0.01 s as a metric for physical performance. The average of two measurements was used for subsequent analyses. For this test, a linear 6 m area was established, with yellow markings being used to mark the 1, 5, and 6 m distances. Adults were then requested to walk at a normal speed from the 0 m starting point to the 6 m mark, with experimental timing beginning when they crossed the 1 m mark and ending when they crossed the 5 m mark. Subjects wore typical shoes during testing and were allowed to use mobility aids, but could not receive assistance from others. There were no time limits, and participants were allowed to pause and rest if desired, although sitting was not permitted.

### Additional measurements

The height and weight of study participants were measured by trained personnel using a standardized approach with a CSTF—5,000 style apparatus (Tongfang Health Technology Co., Ltd.).

### Definition of severe sarcopenia

Severe sarcopenia was defined as per the AWGS2019 diagnostic criteria based on low muscle mass (ASMI, males: < 7.0 kg/m^2^; females: < 5.7 kg/m^2^), low muscle strength (handgrip strength, males: < 28 kg, females: < 18 kg), and poor physical performance (4 m walking test < 1 m/s) (1).

### Ishii test analyses

The odds of sarcopenia were approximated using the Ishii test. In males, Ishii test scores were calculated as follows: 0.62 × (age − 64) − 3.09 × (grip strength − 50) − 4.64 × (calf circumference − 42). In females, Ishii scores were calculated as follows: 0.80 × (age − 64) − 5.09 × (grip strength − 34) − 3.28 × (calf circumference − 42) (18). As the Ishii test has no recommended cut-off values for the diagnosis of severe sarcopenia, the maximum Youden’s index values were used as cut-off values in the present study.

### Statistical analyses

Data were analyzed using SPSS 23.0 (IBM Corp, NY, United States). We used the Paired-Sample Sensitivity Power Analysis from the Diagnostic Tests (ROC) of PASS 11.0 software to calculate the sample size needed for this study. Results suggested that 2,390 samples were enough. Categorical data are given as numbers (percentages), while normally and non-normally distributed continuous data are given as means ± SD and the median and inter-quartile range (IQR), respectively. Data were compared between groups using Student’s *t*-tests, rank-sum tests, and Pearson’s Chi-square test as appropriate. An exclusion test focused on sensitivity and negative predictive value (NPV) together with the area under the ROC curve (AUC) was used in this study to gauge the accuracy of the Ishii test. The Youden index is the sum of sensitivity and specificity minus 1, that is, Youden index = sensitivity + specificity − 1. The calculated Youden index values were then sorted and the point corresponding to the highest value was selected as the optimal cut-off point. The AUC was used to measure the precision of this screening tool, while differences among ROC curves were assessed via the DeLong method (25). The receiver operating characteristic (ROC) curve was then constructed by plotting the sensitivity (y-axis) against the 1-specificity (x-axis) to derive the AUROC. Corresponding specificity, sensitivity, positive predictive value (PPV), and NPV values were then assessed using these cut-off thresholds, with a two-sides *p* < 0.05 as the significance threshold.

## Results

### Study population

In total, this study included 4,177 individuals ≥ 50 years old including 2,668 females (63.9%) and 1,509 males (36.1%). These included 568 (13.6%) participants affected by severe sarcopenia, of whom 237 were male (15.7%) and 331 were female (12.4%). The prevalence of severe sarcopenia among males < 60, 60–69, and ≥ 70 years of age were 3.55%, 13%, and 35.42%, respectively, while among females these respective prevalence rates were 4.43%, 10.69%, and 36.34% (Table 1).

**Table 1:** Study participant characteristics in the severe sarcopenia and non-severe sarcopenia groups.

Characteristics	Male			Female		
	Non-severe sarcopenia	Severe sarcopenia	*P*	Non-severe sarcopenia	Severe sarcopenia	*P*
	(*n* = 1,272)	(*n* = 237)		(*n* = 2,337)	(*n* = 331)	
Age, years, median (iqr)	62(55,68)	68(63,74)	<0.01	60(54,65)	70(62,75)	<0.01
<60, *n* (%)	462(96.45)	17(3.55)		1,122(95.57)	52(4.43)	
60–69, *n* (%)	562(87)	84(13)		919(89.31)	110(10.69)	
≥70, *n* (%)	248(64.58)	136(35.42)		296(63.66)	169(36.34)	
Ethnic			0.001			<0.01
Han, n (%)	474(37.26)	120(50.63)		1,057(45.23)	168(50.76)	
Tibetan, *n* (%)	410(32.23)	51(21.52)		532(22.78)	83(25.08)	
Qiang, *n* (%)	308(24.21)	48(20.25)		620(26.53)	40(12.08)	
Yi, *n* (%)	59(4.64)	13(5.49)		94(4.02)	35(10.57)	
The other, *n* (%)	21(1.66)	5(2.11)		34(1.44)	5(1.51)	
BMI, kg/m^2^, mean (SD)	26.1(3.42)	22.3(3.08)	<0.01	25.93(3.76)	22.11(3.83)	<0.01
Gait speed, m/s, median (iqr)	0.89(0.76,1.01)	0.78(0.66,0.91)	<0.01	0.85(0.71,0.98)	0.7(0.53,0.82)	<0.01
Handgrip strength, kg, mean (SD)	30.33(9.14)	23.72(8.12)	<0.01	19.14(5.46)	13.17(2.97)	<0.01
ASMI, kg/m^2^, median (iqr)	7.6(7.3,8.1)	6.5(6.2,6.8)	<0.01	6.3(5.9,6.8)	5.3(5,5.5)	<0.01
CC, cm, median (iqr)	36.15(34.5,38)	32.7(31.01,34)	<0.01	35(33.05,36.85)	30.55(29.55,32.25)	<0.01
Ishii test score, mean (SD)	85.94(35)	127.19(32.51)	<0.01	95.96(32.97)	146.27(20.8)	<0.01

BMI, body mass index; ASMI, appendicular skeletal muscle index; CC, calf circumference. Non-severe sarcopenia included both subjects with sarcopenia and normal subjects.

Overall, severe sarcopenia prevalence was significantly higher among males relative to females. There were significant differences in age, ethnicity, body mass index (BMI), gait speed, ASMI, CC, hand-grip strength, and Ishii test scores when comparing individuals with and without severe sarcopenia in both the male and female subgroups (Table 1).

### Cut-off points for sarcopenia from Ishii test

**Figure 1:**
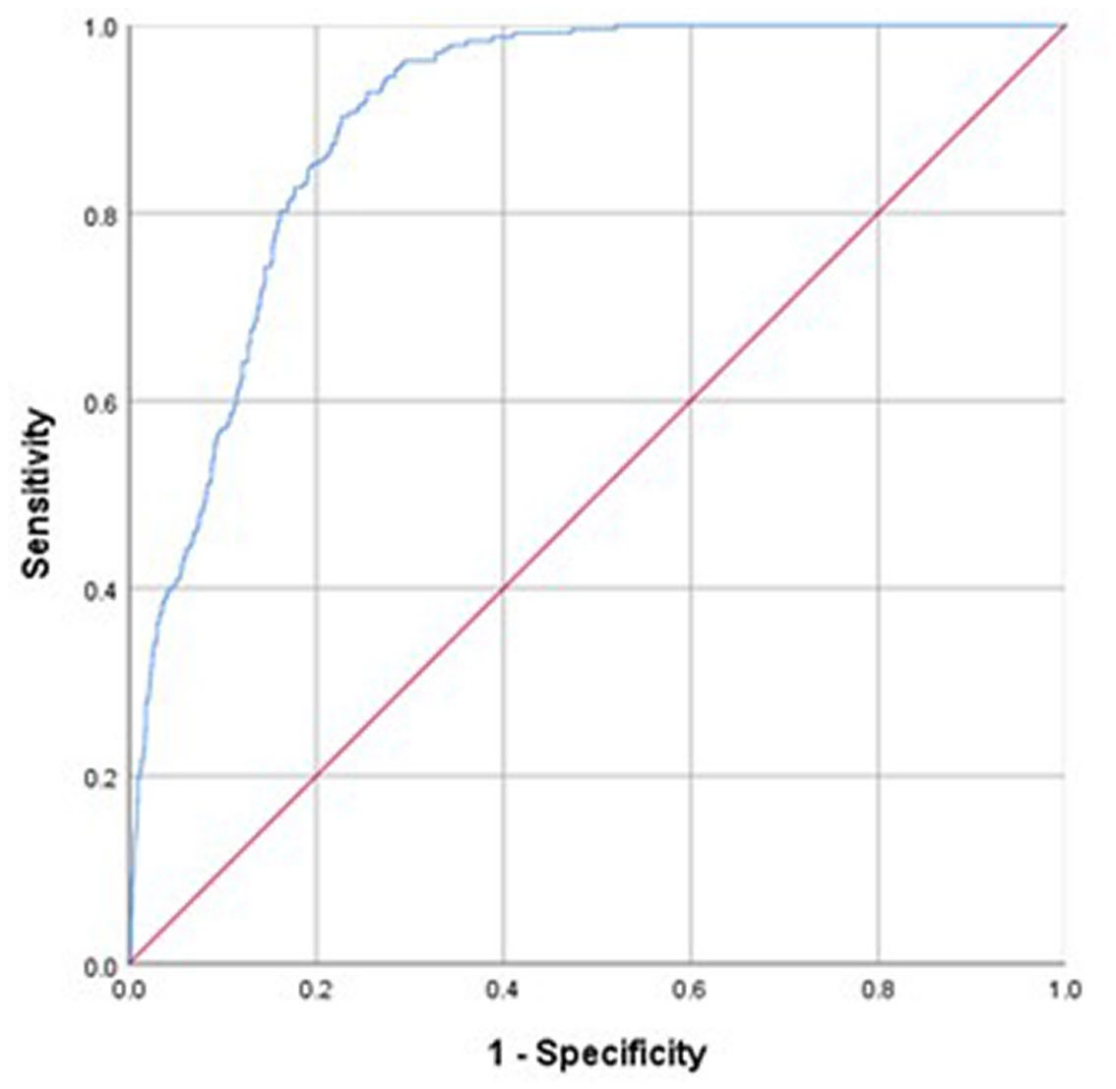
Sensitivity and specificity analyses and ROC models used for Ishii test screening for severe sarcopenia diagnosed as per the AWGS2019 criteria in males.

**Figure 2:**
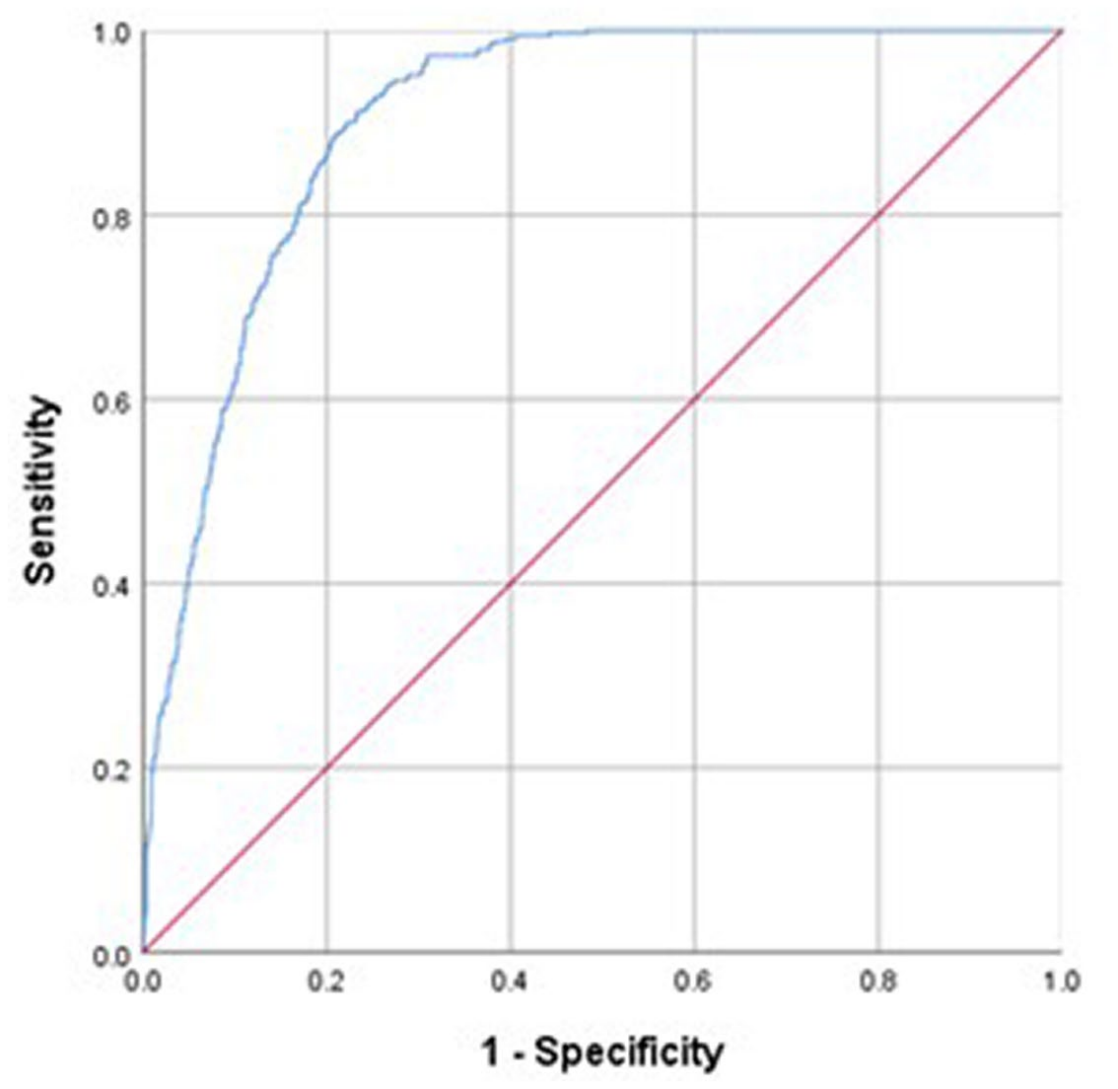
Sensitivity and specificity analyses and ROC models used for Ishii test screening for severe sarcopenia diagnosed as per the AWGS2019 criteria in females.

Overall, the Ishii test was able to effectively predict severe sarcopenia in both females (AUC: 0.905, 95% CI: 0.892–0.917) and males (AUC: 0.899, 95% CI: 0.883–0.916; Figures 1, 2). As no recommended Ishii test cut-off values have been established for use when screening for severe sarcopenia, the maximum Youden’s index values were selected as cut-off thresholds for use in the present study. Import the Ishii score and whether you have sarcopenia into SPSS software, choose to analyze the ROC curve, and then the area under the ROC curve and the “coordinates of the curve” containing a series of sensitivity and 1-specificity values can be obtained. Then calculate the Youden index by the formula Youden index = sensitivity + specificity − 1 in the EXCEL table. Then sort all the Youden indices, and the critical value corresponding to the largest Youden index is the optimal critical value, sensitivity and specificity. The largest Youden index obtained in this study was ≥114 for males and ≥120 for females. Using these cut-offs, the Ishii test yielded respective sensitivity, specificity, PPV, and NPV values of 90.03, 77.05%, 0.36, and 0.98 in females and 89.45%, 77.15%, 0.42, and 0.98 in males (Table 2).

**Table 2:** Sensitivity and specificity analyses and ROC models used for Ishii test screening for severe sarcopenia diagnosed as per the AWGS2019 criteria.

	Sensitivity, %	Specificity, %	PPV	NPV	AUC
**Male**					
Ishii test ≥ 114	89.45	77.15	0.42	0.98	0.899(0.883–0.916)
**Female**					
Ishii test ≥ 120	90.03	77.05	0.36	0.98	0.905(0.892–0.917)

## Discussion

This study is the first to have analyzed the performance of the Ishii test when used to screen for severe sarcopenia among community-dwelling adults in Asia based upon the AWGS2019 consensus criteria. These results offer important evidence in support of the accuracy of the Ishii test as a tool for identifying patients with severe sarcopenia. Overall, 13.6% of community-dwelling participants in this study exhibited severe sarcopenia, with this value being in line with previously reported prevalence rates (2). However, severe sarcopenia prevalence was herein found to be higher among males relative to females, in contrast to previous reports suggesting similar prevalence rates irrespective of gender when measured as per the AWGS2019 criteria (2). This may be attributable to differences in inclusion criteria for this study population or in the criteria used to define severe sarcopenia.

The accuracy of the test depends on how well the test separates the group being tested into those with and without the disease in question. Accuracy is measured by the area under the ROC curve (26). In general, an AUC value > 0.8 is indicative of a high degree of accuracy. The results of the present study revealed the Ishii test to exhibit a high degree of accuracy as a tool for severe sarcopenia screening among community-dwelling Chinese middle-aged and older adults. The AUC value for the Ishii test in this cohort was >0.8 in both males and females, and it exhibited high sensitivity and moderate specificity. Importantly, this high sensitivity rate suggests that the Ishii test can be readily used to identify members of the community at risk of severe sarcopenia.

Optimal Ishii test cut-off values used to identify severe sarcopenia in the present. Study were selected based on maximum Youden’s index value (≥114 for males, ≥120 for females) based on the AWGS2019 reference standard (2). When using these cut-off values as a diagnostic threshold, the Ishii test yielded NPVs of 98% in both males and females in the present study cohort, in line with prior reports indicating the negative predictive utility of this test. In another report, the Ishii test exhibited sensitivity and NPV values of up to 100% when diagnosing severe sarcopenia among adults ≥ 60 years of age in a geriatric outpatient clinic of a university hospital in Turkey using the EWGSOP criteria as a reference gold standard, when using cut-off values recommended by the founder of the Ishii test, namely, >105 for men and >120 for women (19).

In a separate study of the utility of the Ishii test in evaluating 199 nursing home residents in Western China, the respective sensitivity, specificity, PPV, NPV, and AUC values were 89.6%, 83.3%, 0.73, 0.94, 0.891 when utilizing AWGS2019 criteria as a reference gold standard (22). When using the SARC-F and SARC-Calf criteria, a 130-point cut-off threshold has been recommended to screen for severe sarcopenia, with the Ishii test AUC outperforming that of other screening tools under these conditions (22). However, the Ishii test was not compared to other screening or diagnostic tests in the present study, so similar conclusions cannot be drawn for the present patient cohort. Overall, the Ishii test exhibited high sensitivity and accuracy, which may be attributable to the fact that it takes grip strength, which is itself a diagnostic criterion for sarcopenia, into consideration. While grip strength is just one component of the overall diagnosis of sarcopenia, it can be assessed in an inexpensive, convenient, and portable manner in contrast to DXA and BIA, highlighting its potential value as a screening tool. Overall, the results of this study thus suggest that the Ishii test offers value as a means of screening for severe sarcopenia among community-dwelling adults, although additional research will be essential to validate and expand upon these conclusions.

One strength of the present study is the inclusion of a large cohort of middle aged and older adults from western China. There are certain limitations to this study. For one, a BIA approach was used to approximate skeletal muscle mass, rather than gold-standard approaches such as CT, MRI, or DXA. While less accurate than DXA, BIA is inexpensive, does not entail the need for X-ray exposure, and can be more readily utilized to evaluate community-dwelling individuals. Importantly, BIA has been validated (27, 28), and it has been recommended as an alternative approach to establishing muscle mass under the AWGS2019 criteria (1). Secondly, this study only included community-dwelling middle-aged and older adults from Western China. These results may thus not be representative of findings for patients residing in nursing homes or hospitals. Third, this WCHAT study is an ongoing cohort analysis, and only the associated 2018 cross-sectional data were used for the present analysis, precluding any assessment of the predictive validity of the Ishii test. Moreover, we did not assess the associations between Ishii test findings and adverse outcomes. Fourth, we excluded individuals who had not participated in sarcopenia screening or had missing values, which may have influenced the results. Despite these limitations, this was a large-scale multi-center study that was able to adjust properly for confounders in regression analyses, enhancing the reliability of our overall results.

## Conclusion

These findings suggest that the Ishii test represents a promising screening tool and a candidate diagnostic test for severe sarcopenia among community-dwelling older adults in China, with recommended Ishii-test cut-off values of ≥114 for males and ≥120 for females when screening for severe sarcopenia.

## Data availability statement

The original contributions presented in the study are included in the article/supplementary material, further inquiries can be directed to the corresponding authors.

## Ethics statement

The current research was approved by the Ethical Review Committee of West China Hospital of Sichuan University with the committee’s reference number 2017(445). The patients/participants provided their written informed consent to participate in this study.

## Author contributions

SL and XC contributed to conceptualization, data collection, data curation, formal analysis, writing the original draft, and review and editing of the paper. LH and JY contributed to data collection, data curation, and review and editing of the paper. XX contributed to data collection and data curation. JY, BD, and LC contributed to study conceptualization, funding acquisition, investigation, methodology, project administration, supervision, and review and editing of the paper. All authors contributed to the article and approved the submitted version.

## Funding

This work was supported by the National Key R&D Program of China (2018YFC2002400, 2020YFC2005600, 2020YFC2005602, 2018YFC2000305, and 2017YFC0840101), 1.3.5 project for disciplines of excellence, West China Hospital, Sichuan University (ZYGD20010), “Project of Max Cynader Academy of Brain Workstation, WCHSCU” (HXYS19005), and Chengdu Science and Technology Bureau Major Science and Technology Application Demonstration Project” (2019YF0900083SN).

## Conflict of interest

The authors declare that the research was conducted in the absence of any commercial or financial relationships that could be construed as a potential conflict of interest.

## Publisher’s note

All claims expressed in this article are solely those of the authors and do not necessarily represent those of their affiliated organizations, or those of the publisher, the editors and the reviewers. Any product that may be evaluated in this article, or claim that may be made by its manufacturer, is not guaranteed or endorsed by the publisher.

## Abbreviations

AWGS: Asia Working Group of Sarcopenia
WCHAT: West China Health and Aging Trend
PPV: Positive predictive value
NPV: Negative predictive value
AUC: Area under the ROC curve
CT: Computed tomography
MRI: Magnetic-resonance imaging
DXA: Dual-energy X-ray analysis
BIA: bio-Impedance analysis
EWGSOP: European Working Group on Sarcopenia in Older People updated version
ASM: Appendicular skeletal muscle mass
ASMI: The ASM index
IQR: Inter-quartile range
BMI: Body mass index
CC: Calf circumference
CI: Confidence interval
SD: Standard deviation
